# Distinct Polarization of Human Macrophages in Response to Infection with the Periodontitis-Associated Bacteria *Fusobacterium nucleatum*, *Porphyromonas gingivalis*, and *Tannerella forsythia*

**DOI:** 10.3390/cells15212016

**Published:** 2026-11-05

**Authors:** Katharina Schwarz, Carlotta Rickert, Fabian L. Kendlbacher, Oliwia Miłek, Christian Behm, Christina Schäffer, Oleh Andrukhov

**Affiliations:** 1 Competence Center for Periodontal Research, University Clinic of Dentistry, Medical University of Vienna, A-1090 Vienna, Austria; katharina.a.schwarz@meduniwien.ac.at (K.S.); c.rickert96@gmail.com (C.R.); oliwia.milek@meduniwien.ac.at (O.M.); christian.behm@meduniwien.ac.at (C.B.); 2 NanoGlycobiology Research Group, Institute of Biochemistry, Department of Natural Sciences and Sustainable Resources, Universität für Bodenkultur Wien, A-1190 Vienna, Austria

**Keywords:** periodontitis, macrophage polarization, THP-1 macrophages, innate immunity, keystone pathogen, pathobiont

## Abstract

**Highlights:**

**What are the main findings?**

- *P. gingivalis* inhibited both M1 and M2 macrophage polarization and broadly reduced pro- and anti-inflammatory cytokine production.
- *F. nucleatum* and *T. forsythia* promoted a predominantly pro-inflammatory macrophage response, particularly in M1-polarized cells.

**What are the implications of the main findings?**

- Periodontitis-associated bacteria exert distinct species-specific effects on macrophage polarization and inflammatory activity.
- *P. gingivalis* appears to attenuate macrophage inflammatory responses, whereas *F. nucleatum* and *T. forsythia* predominantly promote them.

**Abstract:**

Periodontal health and disease are shaped by a dynamic interaction between the subgingival microbiome and the host immune system, in which gingival macrophages contribute to tissue homeostasis, disease progression, and inflammation resolution. Here, we investigated how three periodontitis-associated bacteria, *Fusobacterium nucleatum*, *Porphyromonas gingivalis*, and *Tannerella forsythia*, shape the polarization of human macrophages in vitro. THP-1 monocytes were differentiated into unpolarized M0 macrophages, or polarized toward the M1 or M2 phenotype, and infected with each bacterium at different multiplicities of infection. Polarization was assessed from the surface expression of CD80 (M1) and CD209 (M2) and from pro- and anti-inflammatory cytokine production, measured by a multiplex assay. The response to *P. gingivalis* differed strikingly from the two other species. *Porphyromonas gingivalis* suppressed both M1 and M2 polarization and reduced the levels of several pro- and anti-inflammatory cytokines. In contrast, *F. nucleatum* and *T. forsythia* enhanced CD80 expression and pro-inflammatory cytokine production in M1 macrophages and modestly increased pro-inflammatory mediators in M2 macrophages. These findings point to a distinct role for *P. gingivalis* as a keystone pathogen that subverts the innate immune response, and for *F. nucleatum* and *T. forsythia* as periodontal pathobionts.

## 1. Introduction

Periodontitis is a highly prevalent chronic inflammatory disease of the tooth-supporting apparatus that destroys periodontal tissues and, if left untreated, leads to tooth loss [1,2]. Its etiology is complex and not fully understood, but the transition from health to disease is widely attributed to a disruption of the symbiotic relationship between the subgingival microbiota and the host immune system, resulting in a dysbiotic state [3,4]. This shift is characterized by a reorganization of the subgingival microbial community together with a dysregulated host response, and it is promoted by a range of environmental and host risk factors [5].

The microbiology of periodontal disease is intricate, and our understanding of it has advanced alongside improvements in analytical techniques and computational methods [6]. Early studies using DNA-DNA hybridization identified several bacterial complexes that predominated at the different stages of biofilm maturation and disease progression [7,8]. The so-called “red complex,” comprising *Porphyromonas gingivalis*, *Tannerella forsythia*, and *Treponema denticola*, was long regarded as the principal etiological factor in periodontitis [9]. Subsequent next-generation sequencing revealed a far more complex subgingival microbiota encompassing hundreds of species with distinct community structures in health and disease [3,10]. Periodontitis is therefore now viewed as a polymicrobial infection driven by a complex subgingival microbial community rather than by any single species [11]. Many community members act as pathobionts, triggering or amplifying inflammation once dysbiosis sets in or when specific risk factors are present [12].

Deciphering the interplay between the subgingival microbiome and the host immune system is crucial for understanding both the mechanisms underlying the maintenance of oral health and the progression of periodontitis. Although periodontitis is a polymicrobial infection, characterizing how individual species engage the immune system remains a necessary step toward unravelling the complex host-microbiome interactions within the subgingival pocket. Several community members have been studied in this context. Most attention has focused on *P. gingivalis*, regarded as a keystone pathogen because of its capacity to subvert host immunity and orchestrate the formation of a dysbiotic community [13,14]. Another “red-complex” member, *T. forsythia*, possesses a range of virulence factors and engages in complex interactions with the host immune system [15,16]. *Fusobacterium nucleatum*, a central constituent of the oral microbial community and subgingival biofilm, has traditionally been considered a bridging species that links early colonizers such as streptococci and *Actinomyces* species with late colonizers such as *P. gingivalis* and *T. forsythia* [17,18]. More recently, this view has been revised, and *F. nucleatum* is now recognized as a key inflammation-promoting pathobiont associated with periodontitis and several extraoral diseases [19].

Macrophages are innate immune cells that participate in numerous processes relevant to periodontitis, including phagocytosis, antigen presentation, efferocytosis, inflammation resolution, and tissue regeneration [20,21,22]. They are highly heterogeneous and can be broadly divided into pro-inflammatory M1 and anti-inflammatory M2 phenotypes [23]. These phenotypes differ in surface marker expression and cytokine output: M1 macrophages express high levels of CD80 and secrete tumor necrosis factor (TNF)-α, interferon (IFN)-γ, interleukin (IL)-6, whereas M2 macrophages express CD209 and produce high levels of IL-10 and arginase [23,24,25]. Importantly, macrophages exhibit a high degree of plasticity and typically combine features of both states, which represent extremes of a spectrum [24]. In periodontitis, macrophages play multiple roles, adapting their phenotype to the periodontal microenvironment to regulate both tissue damage and repair [20,26].

Several studies have examined how periodontitis-associated bacteria influence macrophage polarization, most focusing on *P. gingivalis*. In THP-1 macrophages, *P. gingivalis* has been reported to promote M1 and inhibit M2 polarization [27] and to increase the production of pro-inflammatory cytokines, particularly IL-1β, TNF-α, and IL-6 [28]. It similarly induced pro-inflammatory cytokine production in human monocyte-derived macrophages [29]. In contrast, a recent study reported that *P. gingivalis* suppresses the IFN-γ-induced response in human THP-1 macrophages [30]. Studies in mouse macrophages likewise showed that *P. gingivalis* induces pro-inflammatory cytokine production, promotes M1 polarization, and inhibits M2 polarization [31,32,33,34]. The interaction of *P. gingivalis* with macrophages has been implicated not only in periodontitis but also in systemic conditions such as atherosclerosis, rheumatoid arthritis, and Alzheimer’s disease [35]. By comparison, the effects of *F. nucleatum* and *T. forsythia* on macrophage polarization have been investigated far less, particularly in human cells. One study showed that *F. nucleatum*, co-infected with *T. forsythia*, activates NF-kB signaling in human macrophages and pro-inflammatory cytokine production in mouse macrophages [36]. Other reports indicate that *F. nucleatum* drives M2 polarization in mouse macrophages through a TLR-4-dependent mechanism [37,38]. In THP-1 and primary human macrophages, various *T. forsythia* clinical isolates induced both pro- and anti-inflammatory cytokines [39], and our own work demonstrated that *T. forsythia* elicits pro-inflammatory cytokine production in U937 human macrophages [40,41,42]. Overall, the effects of the different periodontitis-associated bacteria on macrophage polarization remain incompletely defined.

The present study aimed to determine how three periodontitis-associated species, *F. nucleatum*, *P. gingivalis*, and *T. forsythia*, affect the polarization of human macrophages under a range of in vitro conditions. Using the human monocytic cell line THP-1, we differentiated the cells into unpolarized macrophages (M0) and infected them with each bacterium at different multiplicities of infection (MOIs). In parallel, M0 macrophages were polarized toward the pro-inflammatory M1 or anti-inflammatory M2 phenotype while being simultaneously infected. Polarization was assessed from the surface expression of characteristic markers and the production of signature cytokines. Our data reveal marked differences in the capacity of these periodontitis-associated bacteria to modulate macrophage polarization and cytokine production.

## 2. Materials and Methods

### 2.1. Cultivation of THP-1 Cells

The immortalized human monocytic cell line THP-1 (ATCC, Manassas, VA, USA), was used in the present study. After purchase, THP-1 cells were expanded and stored at −80 °C until use. Immediately after thawing, THP-1 cells were cultured in RPMI-1640 medium (Capricorn Scientific, Ebsdorfergrund, Germany) supplemented with 20% fetal bovine serum (FBS, Capricorn Scientific, Ebsdorfergrund, Germany) at 37 °C and 5% CO_2_ in a humidified atmosphere. Once exponential growth began, the FBS concentration was reduced to 10%. Cell density was maintained between 1 × 10^5^–10^6^ cells/mL throughout cultivation. Cultures were tested periodically for mycoplasma, and THP-1 cells at passages 7–20 were used for experiments.

### 2.2. Cultivation of Bacteria

Bacteria were cultivated as described in our previous studies [43,44]. *Tannerella forsythia* ATCC 43037 (American Type Culture Collection, Manassas, VA, USA) was cultured anaerobically at 37 °C for 5 days on Fastidious Anaerobe agar (FA; Neogen, Lansing, MI, USA) supplemented with 5% horse blood (Sigma–Aldrich, St. Louis, MO, USA) and 20 μg/mL *N*-acetylmuramic acid (NAMA; Sigma–Aldrich, St. Louis, MO, USA) [16,45]. *Fusobacterium nucleatum* subsp. *nucleatum* KP-F8 (OMZ598) and *Porphyromonas gingivalis* ATCC 33277 were grown anaerobically at 37 °C for 5 days on Columbia Blood Agar (CBA; Oxoid, Altrincham, UK) supplemented with 5% horse blood [18,41,46,47].

Before each experiment, bacteria were collected by gently scraping the agar plates with a cell scraper and transferring the material into a tube containing 1 mL of phosphate-buffered saline (PBS). After centrifugation, bacterial cell pellets were resuspended in RPMI-1640 supplemented with 3% FBS. The optical density at 600 nm (OD_600_) was measured with an Ultrospec 10 cell density meter (Biochrom, Cambridge, UK) and adjusted to 1.0 by dilution with medium. For each bacterial species, the relationship between an OD_600_ of 1.0 and colony-forming units (CFU) per milliliter of culture was determined by dilution plating and colony counting, with three biological and three technical replicates as previously described [41]. An OD_600_ of 1.0 corresponded to 3 × 10^8^ CFU for *T. forsythia*, 1 × 10^9^ CFU for *F. nucleatum*, and 1 × 10^9^ CFU for *P. gingivalis*.

### 2.3. Differentiation, Polarization and Bacterial Infection of THP-1 Monocytes

THP-1 cells were seeded in 6-well plates at a concentration of 2.2 × 10^5^ cells/well in 2 mL RPMI-1640 supplemented with 3% FBS and stimulated with 10 ng/mL phorbol-12-myristate-13-acetate (PMA, Sigma-Aldrich, St. Louis, MO, USA) for 72 h. This was followed by a 24 h resting period in culture media without PMA. The resulting adherent cells were considered M0 macrophages. To polarize these M0 macrophages toward the M1 or M2 phenotype, cells were treated for 48 h with either 1 µg/mL of *Escherichia coli* lipopolysaccharide (LPS, Invivogen, San Diego, CA, USA) plus 10 ng/mL of IFN-γ (PeproTech, London, UK) or with 20 ng/mL of IL-4 (PeproTech, London, UK) plus 20 ng/mL of IL-13 (PeproTech, London, UK), respectively.

M0 macrophages were infected immediately after differentiation with one of the following bacteria: *F. nucleatum* at an MOI of 50, 100, or 500; *P. gingivalis* at an MOI of 25, 50, or 100; *T. forsythia* at an MOI of 25, 50, or 100. The higher MOI range used for *F. nucleatum* was selected based on preliminary dose-finding experiments, in which an increased proportion of CD36^+^CD80^+^ macrophages was observed at MOI 50 but not at MOI 25. Infected cells were cultured in RPMI-1640 medium supplemented with 3% FBS in a humidified atmosphere at 37 °C and 5% CO_2_ for 48 h. M1 and M2 macrophages were infected with the same bacteria at the same MOIs during their 48 h polarization. M0, M1, and M2 macrophages without bacteria served as controls. At the end of the infection period, conditioned media were collected, centrifuged, and stored at −80 °C in 0.5 mL aliquots, while macrophage viability and surface marker expression were analyzed by flow cytometry.

### 2.4. Analysis of Macrophage Viability and Surface Marker Expression

Attached cells were washed with 1 mL of PBS per well and incubated in 0.5 mL of Accutase cell detachment solution (Capricorn Scientific, Ebsdorfergrund, Germany) for 5 min at 37 °C to promote cell detachment. Cells were collected, washed once with PBS, and resuspended in 1 mL of PBS. Then, 0.5 µL of Fixable Viability Dye eFluor780 (FVD, Invitrogen, Carlsbad, CA, USA; Cat. Nr. 65-0865-14) was added to 1 mL of cell suspension, and cells were incubated for 30 min at 4 °C. Cells were then washed with PBS and resuspended in 90 µL of flow cytometry buffer (PBS supplemented with 3% bovine serum albumin and 0.09% NaN_3_); 10 µL of anti-human Fc-receptor antibodies (Invitrogen, Waltham, MA, USA, Cat. Nr. 14-9161-73) were added for Fc-receptor blocking, and cells were incubated for a further 20 min at 4 °C.

Cells were then stained for 30 min at 4 °C with the following surface marker antibodies at a 1:20-dilution (all from Invitrogen, Waltham, MA, USA): anti-human CD-80 antibody (clone B7-1) conjugated with Super Bright 436 (Cat. Nr. 67-0809-42); anti-human CD36 antibody (clone TR9) conjugated with phycoerythrin (Cat. Nr. MA1-19772); anti-human CD209 antibody (clone eB-h209) conjugated with allophycocyanin (Cat. Nr. 17-2099-42). After staining, cells were washed twice with flow cytometry buffer, resuspended in 500 µL of flow cytometry buffer, and analyzed by flow cytometry.

Flow cytometry was performed on an Attune™ NxT flow cytometer (Thermo Fisher Scientific, Waltham, MA, USA) equipped with violet (405 nm), blue (488 nm), and red (638 nm) lasers; in total, 20,000 events per sample were analyzed. Data were analyzed using FCS Express^TM^ 7 (De Novo Software, Pasadena, CA, USA); the gating strategy is shown in Supplementary Figure S1. Cells were first gated for singlets using Side Scatter Area (SSC-A) versus Side Scatter Width (SSC-W) (Supplementary Figure S1A), and cell debris was excluded by gating Forward Scatter (FSC) against Side Scatter (SSC) (Supplementary Figure S1B). FVD eFluor780 was used to discriminate between live and dead cells (Supplementary Figure S1C). Successfully matured macrophages were identified by gating on CD36^+^ events (Supplementary Figure S1D), which were then further gated as either CD36^+^CD80^+^ events or CD36^+^CD209^+^ events (Supplementary Figure S1E,F), reflecting M1 and M2 polarization, respectively.

Unstained, uninfected M0 macrophages served as negative controls for autofluorescence. FVD was compensated using the ArC^TM^ Amine Reactive Compensation Bead Kit (Invitrogen, Waltham, MA, USA; Cat. Nr. A10346); CD36-PE, CD209-APC, and CD80-Super Bright 702 were compensated using AbC^TM^ Total Antibody Compensation Bead Kit (Invitrogen, Waltham, MA, USA; Cat. Nr. A10497), both according to the manufacturer’s instructions. Marker-specific positivity thresholds were determined using fluorescence-minus-one controls, each containing all staining reagents except the antibody corresponding to the marker being evaluated.

### 2.5. Analysis of Cytokine Production by Multiplex Assay

Cytokine content in the supernatants was analyzed using the LEGENDplex HU Macrophage/Microglia panel (BioLegend, San Diego, CA, USA; Cat. Nr. 740503). The following cytokines were analyzed in V-bottom plates according to the manufacturer’s instructions: the pro-inflammatory TNF-α (limit of detection [LOD]: 2.1 pg/mL), IL-1β (LOD: 2.7 pg/mL), IL-6 (LOD: 1.7 pg/mL), IFN-γ (LOD: 2.9 pg/mL), IFN-γ-induced protein (IP)-10 (LOD: 3.6 pg/mL), IL-23 (LOD: 4.4 pg/mL), IL-12p40 (LOD: 11.8 pg/mL), and IL-12p70 (LOD: 1.2 pg/mL), and the anti-inflammatory cytokines IL-4 (LOD: 2.1 pg/mL), IL-10 (LOD: 1.47 pg/mL), arginase (LOD: 256.5 pg/mL), thymus and activation-regulated cytokine (TARC) (LOD: 2.9 pg/mL), and IL-1 receptor antagonist (IL-1Ra) (LOD: 107 pg/mL).

Fluorescence-encoded beads were analyzed on the Attune™ NxT flow cytometer (Thermo Fisher Scientific, Waltham, MA, USA), with the photomultiplier tube voltages set according to the supplier’s instructions. A total of 300 beads were acquired per analyte, yielding approximately 4000 beads after exclusion of debris. Data were analyzed using the LEGENDplex™ data analysis software 2026-07-01 (BioLegend, San Diego, CA, USA) to calculate the cytokine concentrations in pg/mL. Values below the LOD were classified as undetectable and plotted as 0 pg/mL.

### 2.6. Statistical Analysis

Five independent repetitions were performed for each experimental condition (M0, M1 polarization, and M2 polarization). Differences between groups within each experiment were assessed by repeated-measures analysis of variance with the LSD post hoc test. Differences with *p* < 0.05 were considered statistically significant; exact *p*-values are provided in Supplementary File S1. All statistical analyses were performed using SPSS Statistics 30.0 (IBM, Armonk, NY, USA), and the graphs were generated using GraphPad Prism 11 (GraphPad Software, Boston, MA, USA).

## 3. Results

### 3.1. Effect of Bacterial Infection on the Viability of THP-1 Macrophages

**Figure 1:**
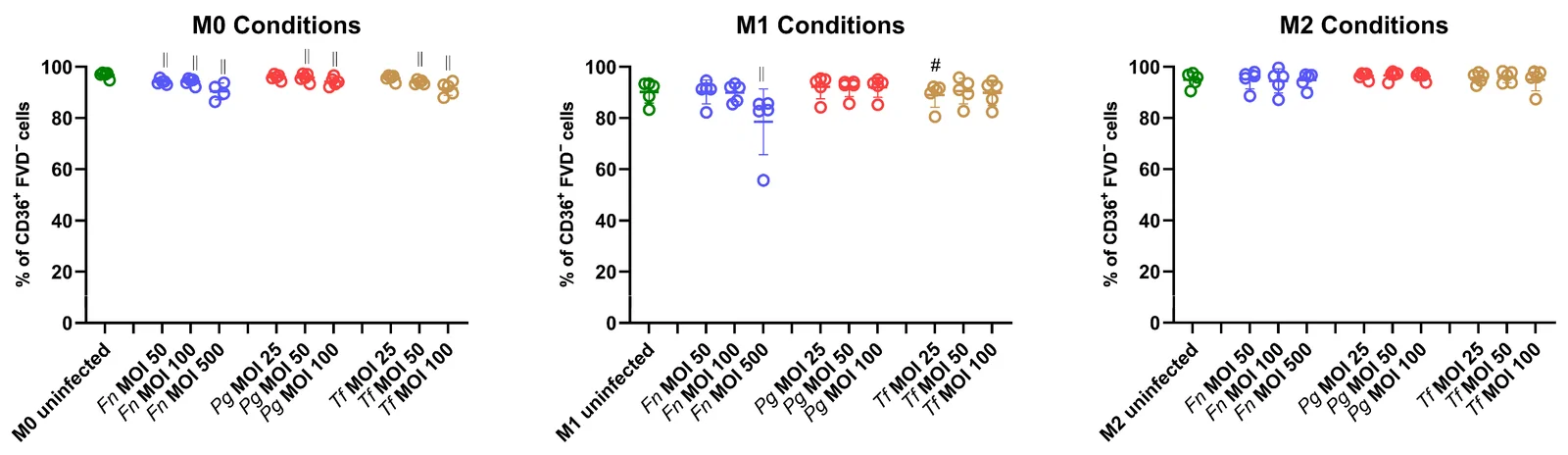
Effect of various bacteria on the viability of macrophages under different experimental conditions. THP-1 monocytes were differentiated into M0 macrophages by PMA treatment and infected with *F. nucleatum* (*Fn*), *P. gingivalis* (*Pg*), or *T. forsythia* (*Tf*) at different MOIs for 48 h (M0 conditions). Some M0 macrophages were further polarized to M1 or M2 macrophages by treatment with IFN-γ and LPS or IL-4 and IL-13, respectively, while being simultaneously infected with different bacteria for 48 h (M1 and M2 conditions, respectively). Cell viability was assessed by flow cytometry based on staining with anti-CD36-PE antibody and FVD eFluor780. The y-axes show the percentage of CD36^+^FVD^−^ events, which were interpreted as the percentage of viable macrophages. Each data point represents an individual experiment; lines and error bars indicate the mean and SD, respectively. Symbols indicate statistically significant differences (*p* < 0.05). ‖, significantly decreased % of CD36^+^FVD^−^ events compared with the respective uninfected control; **#**, significantly decreased % of CD36^+^FVD^−^ events compared with infection with *P. gingivalis* at a similar MOI. Exact *p*-values for all comparisons are provided in Supplementary File S1.

The viability of THP-1 macrophages after infection with each bacterium under the various experimental conditions is shown in Figure 1. In M0 macrophages, infection with all three bacteria caused a slight decrease in the proportion of viable cells, with no differences among species. In M1 macrophages, only *F. nucleatum* at an MOI of 500 significantly reduced viability. In M2 macrophages, bacterial infection had no effect on viability.

### 3.2. Effect of Bacterial Infection on the Characteristic Surface Marker Expression and Cytokine Production in M0 Macrophages

**Figure 2:**
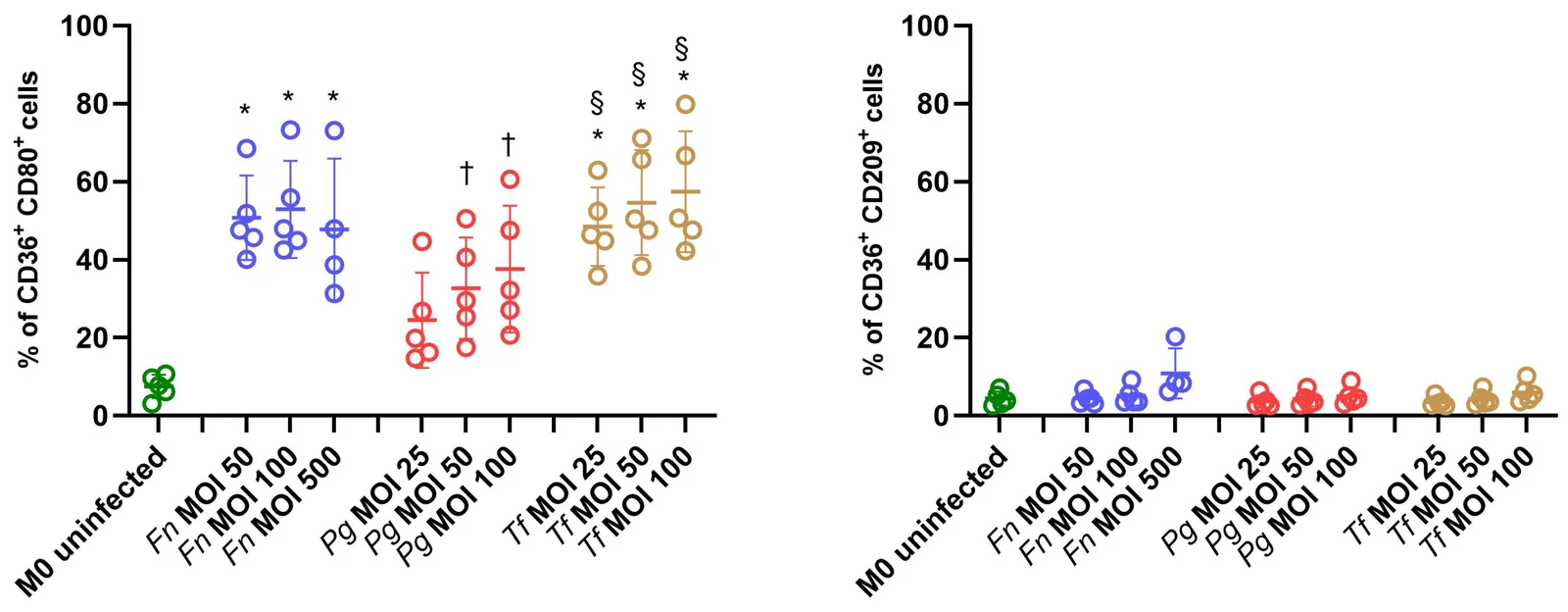
Surface marker expression of M0 macrophages upon bacterial infection. THP-1 monocytes were differentiated into M0 macrophages by PMA treatment and infected with *F. nucleatum* (*Fn*), *P. gingivalis* (*Pg*), or *T. forsythia* (*Tf*) at different MOIs for 48 h. Expression of the characteristic M1 and M2 surface markers CD80 and CD209, respectively, was analyzed by flow cytometry; staining against CD36 confirmed macrophage differentiation. Y-axes show the percentage of CD36^+^CD80^+^ cells (**left panel**) and of CD36^+^CD209^+^ cells (**right panel**), corresponding to M1 and M2 macrophages, respectively. Each data point represents an individual experiment; lines and error bars indicate the mean and SD, respectively. Symbols indicate statistically significant differences (*p* < 0.05). *, significantly increased % of CD36^+^CD80^+^ cells compared with the uninfected control; †, significantly decreased % of CD36^+^CD80^+^ cells compared with *F. nucleatum* infection at a similar MOI; §, significantly increased % of CD36^+^CD80^+^ cells compared with *P. gingivalis* infection at a similar MOI. Exact *p*-values for all comparisons are provided in Supplementary File S1.

The surface expression of CD80 and CD209 in THP-1 macrophages infected under M0 conditions is shown in Figure 2. The proportion of CD80^+^ macrophages increased with all three bacteria in a concentration-dependent manner, although the effect reached statistical significance only for *F. nucleatum* and *T. forsythia*. The proportion of CD80^+^ macrophages induced by these two species was also significantly higher than that induced by *P. gingivalis* at comparable MOIs. None of the infections significantly altered the proportion of CD209^+^ macrophages under these conditions.

**Figure 3:**
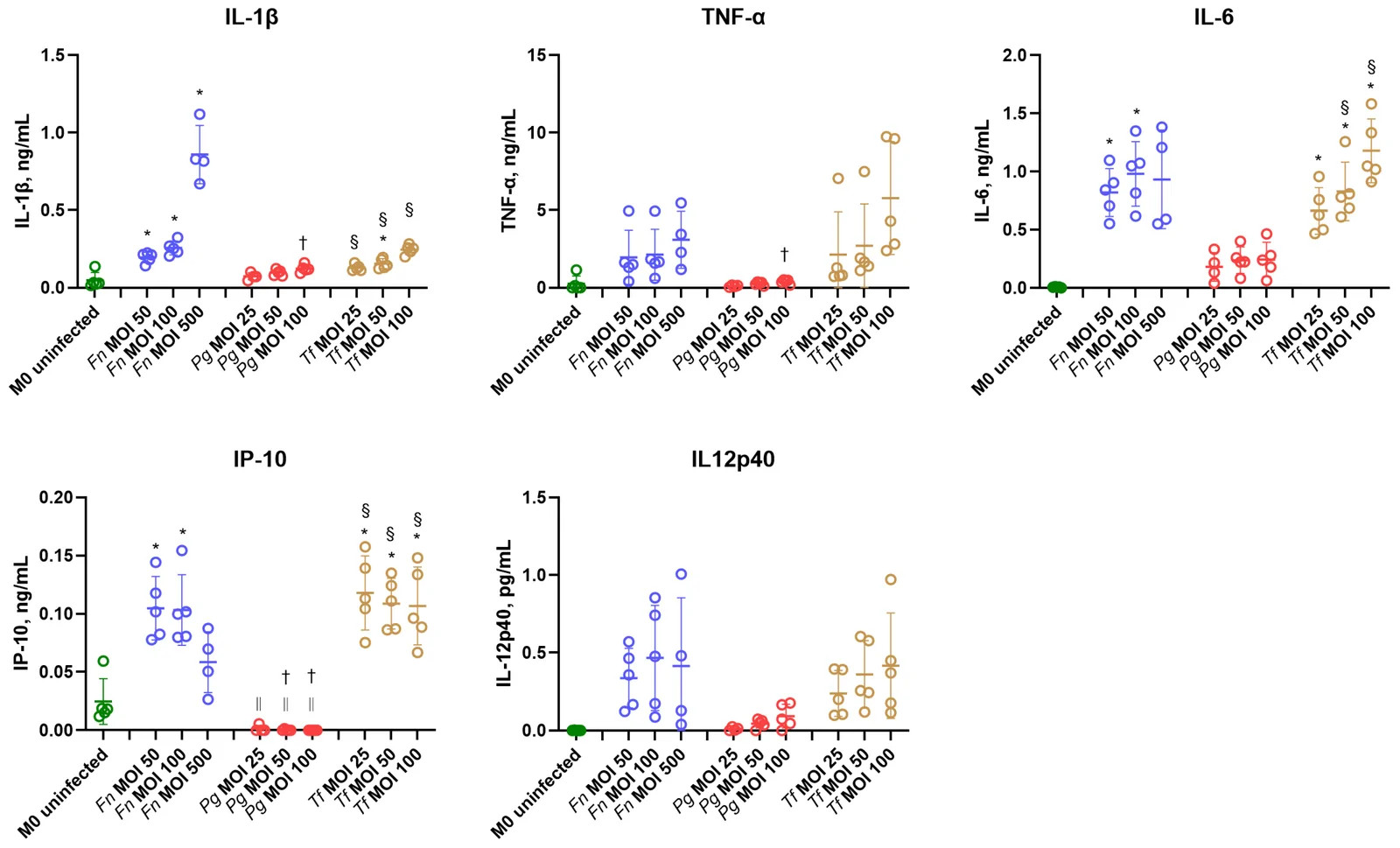
Pro-inflammatory cytokine production by M0 macrophages upon bacterial infection. THP-1 monocytes were differentiated into M0 macrophages by PMA treatment and then infected with *F. nucleatum* (*Fn*), *P. gingivalis* (*Pg*), or *T. forsythia* (*Tf*) at different MOIs for 48 h. Levels of cytokines characteristic of M1 macrophages—IL-1β, TNF-α, IL-6, IP-10, and IL-12p40—in conditioned media were determined by a multiplex assay. Y-axes show the corresponding cytokine concentration in conditioned media. Each data point represents an individual experiment; lines and error bars indicate the mean and SD, respectively. Symbols indicate statistically significant differences (*p* < 0.05). *, significantly increased level of corresponding cytokine compared with the uninfected control; ‖, significantly decreased level of corresponding cytokine compared with the uninfected control; †, significantly decreased level of corresponding cytokine compared with *F. nucleatum* infection at a similar MOI; §, significantly increased level of corresponding cytokine compared with *P. gingivalis* infection at a similar MOI. Exact *p*-values for all comparisons are provided in Supplementary File S1.

The production of pro-inflammatory cytokines by M0 macrophages upon bacterial infection is shown in Figure 3. Infection with *F. nucleatum* and *T. forsythia* generally increased the production of characteristic M1 cytokines, reaching significance for IL-1β, IL-6, and IP-10, but not for TNF-α or IL-12p40. *Porphyromonas gingivalis* significantly decreased IP-10 levels while leaving the other cytokines unchanged, and its overall cytokine induction was consistently lower than that of *F. nucleatum* and *T. forsythia* at comparable MOIs. IFN-γ, IL-23, and IL-12p70 remained below the assay’s detection limit under these conditions.

**Figure 4:**
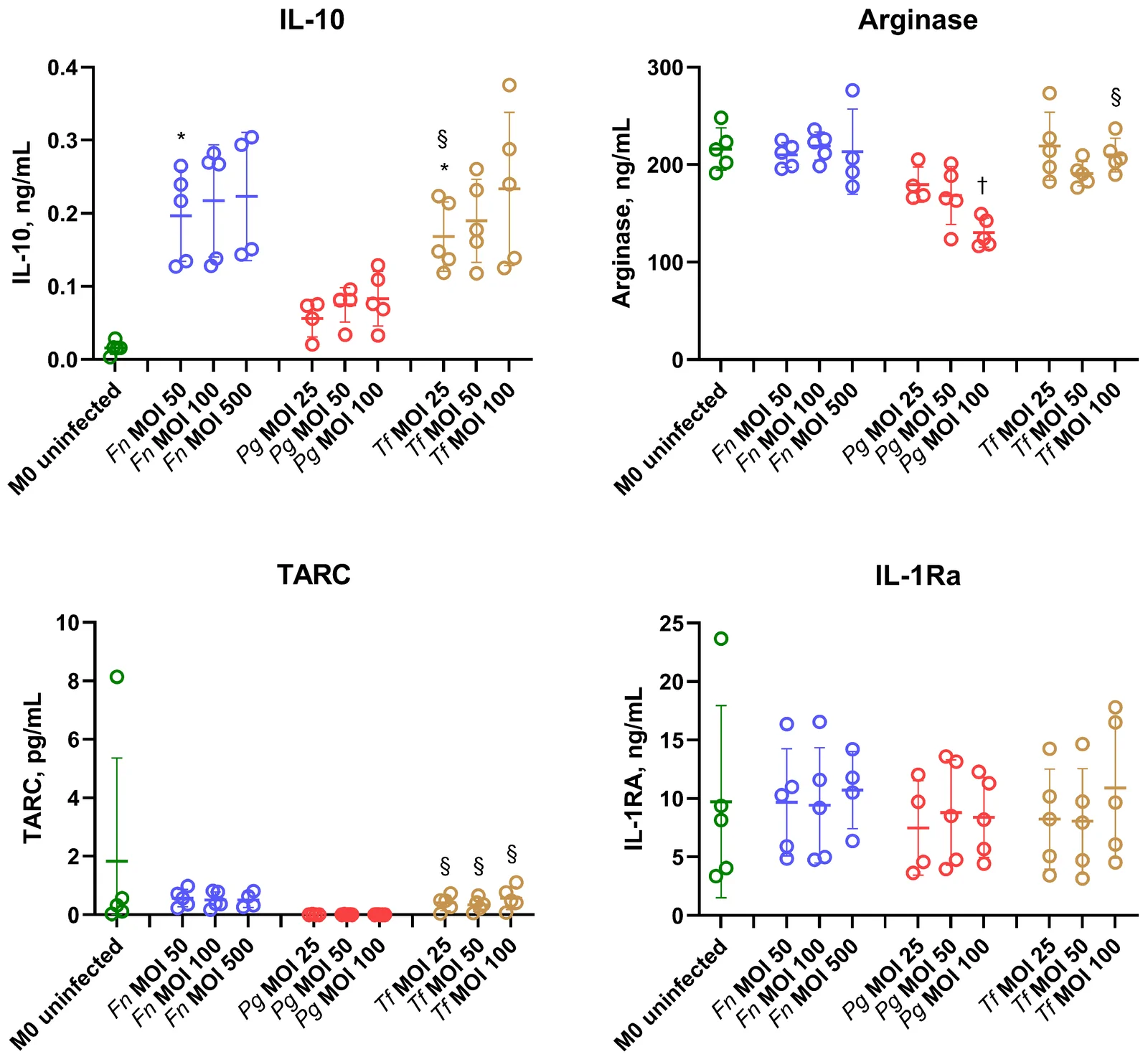
Anti-inflammatory cytokine production by M0 macrophages upon bacterial infection. THP-1 monocytes were differentiated into M0 macrophages by PMA treatment and then infected with *F. nucleatum* (*Fn*), *P. gingivalis* (*Pg*), or *T. forsythia* (*Tf*) at different MOIs for 48 h. The levels of cytokines characteristic of M2 macrophages—IL-10, arginase, TARC, and IL-1Ra—in conditioned media were determined by a multiplex assay. Y-axes show the corresponding cytokine concentration in conditioned media. Each data point represents an individual experiment; lines and error bars indicate the mean and SD, respectively. Symbols indicate statistically significant differences (*p* < 0.05). *, significantly increased level of corresponding cytokine compared with the uninfected control; †, significantly decreased level of corresponding cytokine compared with *F. nucleatum* infection at a similar MOI; §, significantly increased level of corresponding cytokine compared with *P. gingivalis* infection at a similar MOI. Exact *p*-values for all comparisons are provided in Supplementary File S1.

The production of anti-inflammatory cytokines by M0 macrophages upon bacterial infection is shown in Figure 4. *Fusobacterium nucleatum* and *T. forsythia* increased IL-10 production, whereas arginase, TARC, and IL-1Ra were unaffected. *Porphyromonas gingivalis* had no significant effect on any cytokine measured, although arginase and TARC tended to increase. At some MOIs, IL-10, arginase, and TARC levels in *P. gingivalis*-infected M0 macrophages were significantly lower than those induced by *T. forsythia*. IL-4 remained below the assay’s detection limit under these conditions.

### 3.3. Effect of Bacterial Infection on the Characteristic Surface Marker Expression and Cytokine Production in M1 Macrophages

**Figure 5:**
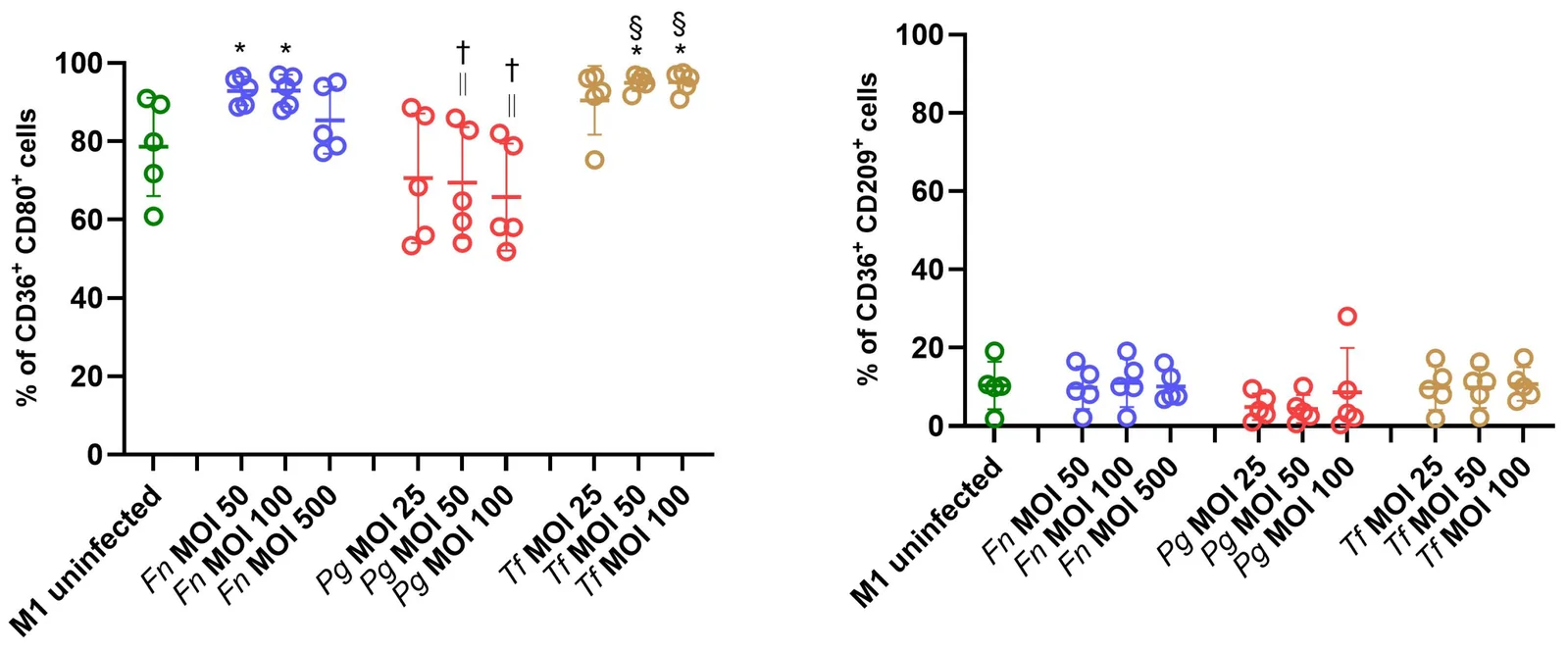
Surface marker expression of M1 macrophages upon bacterial infection. THP-1 monocytes were differentiated into M0 macrophages by PMA treatment, then polarized toward the M1 phenotype by treatment with IFN-γ and *E. coli* LPS while being simultaneously infected with *F. nucleatum* (*Fn*), *P. gingivalis* (*Pg*), or *T. forsythia* (*Tf*) at different MOIs for 48 h. Expression of the characteristic M1 and M2 surface markers CD80 and CD209, respectively, was analyzed by flow cytometry; staining for CD36 confirmed macrophage differentiation. Y-axes show the percentage of CD36^+^CD80^+^ cells (**left panel**) and of CD36^+^CD209^+^ cells (**right panel**), corresponding to M1 and M2 macrophages, respectively. Each data point represents an individual experiment; lines and error bars indicate the mean and SD, respectively. Symbols indicate statistically significant differences (*p* < 0.05). *, significantly increased % of CD36^+^CD80^+^ cells compared to the uninfected control; ‖, significantly decreased % of CD36^+^CD80^+^ cells compared with the uninfected control; †, significantly decreased % of CD36^+^CD80^+^ cells compared with *F. nucleatum* infection at a similar MOI; §, significantly increased % of CD36^+^CD80^+^ cells compared with *P. gingivalis* infection at a similar MOI. Exact *p*-values for all comparisons are provided in Supplementary File S1.

The surface expression of CD80 and CD209 in THP-1 macrophages infected during M1 polarization is shown in Figure 5. *Fusobacterium nucleatum* and *T. forsythia* increased the proportion of CD80^+^ macrophages at MOIs of 50 and 100. In contrast, *P. gingivalis* at comparable MOIs decreased the proportion of CD80^+^ macrophages relative to both the uninfected control and the other two bacteria. Notably, *F. nucleatum* at an MOI of 500 had no significant effect on the proportion of CD80^+^ cells. None of the infections significantly altered the proportion of CD209^+^ macrophages during M1 polarization.

**Figure 6:**
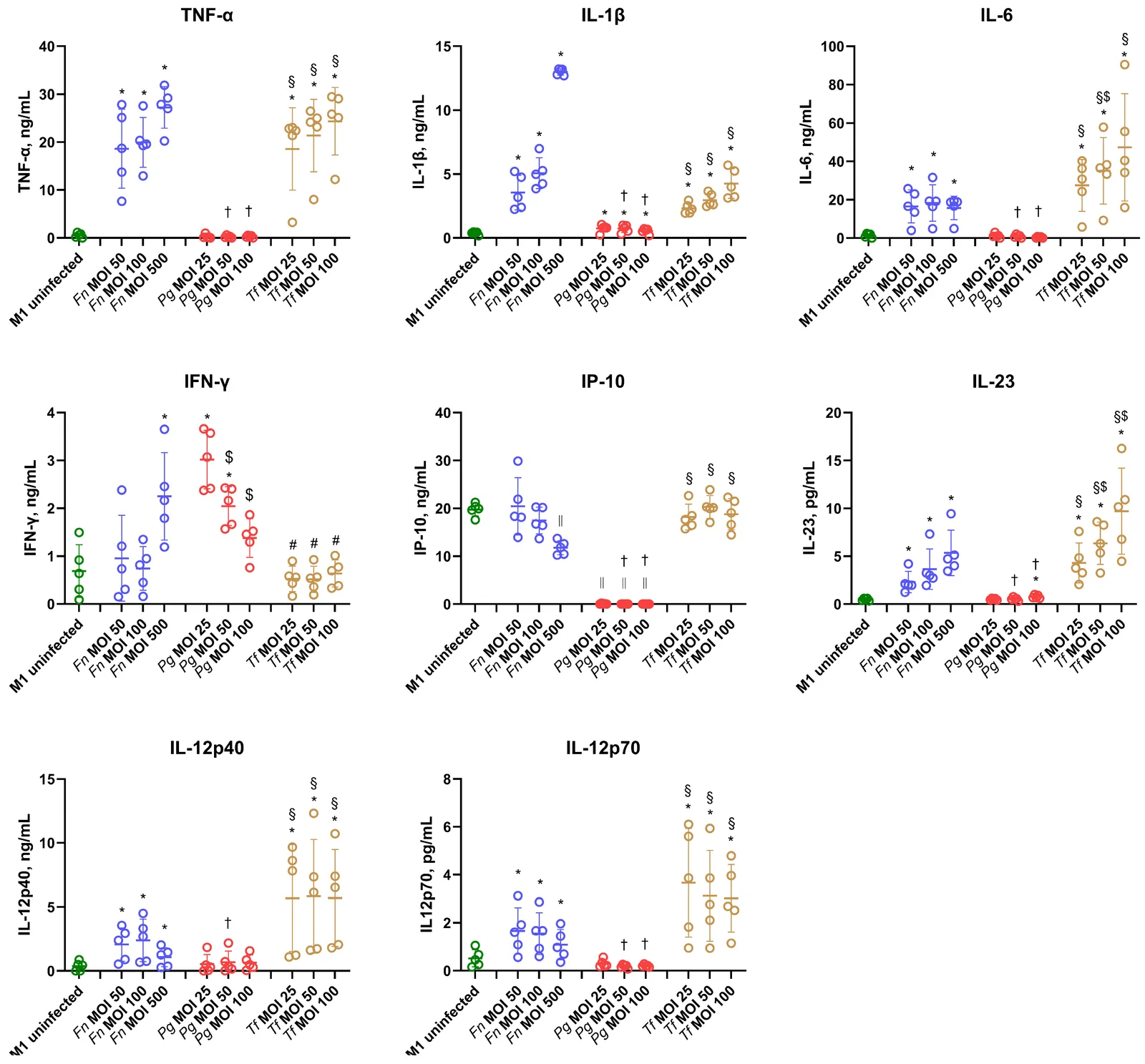
Pro-inflammatory cytokine production by M1 macrophages upon bacterial infection. THP-1 monocytes were differentiated into M0 macrophages by PMA treatment, then polarized toward the M1 phenotype by treatment with IFN-γ and *E. coli* LPS while being simultaneously infected with *F. nucleatum* (*Fn*), *P. gingivalis* (*Pg*), or *T. forsythia* (*Tf*) at different MOIs for 48 h. Levels of cytokines characteristic of M1 macrophages—IL-1β, TNF-α, IL-6, IP-10, and IL-12p40—in conditioned media were determined by multiplex assay. Y-axes show the corresponding cytokine concentration in the conditioned media. Each data point represents an individual experiment; lines and error bars indicate the mean and SD, respectively. Symbols indicate statistically significant differences (*p* < 0.05). *, significantly increased level of corresponding cytokine compared to the uninfected control; ‖, significantly decreased level of corresponding cytokine compared to the uninfected control; †, significantly decreased level of corresponding cytokine compared with *F. nucleatum* infection at a similar MOI; $, significantly increased level of corresponding cytokine compared to *F. nucleatum* infection at similar MOI; §, significantly increased level of corresponding cytokine compared with *P. gingivalis* infection at a similar MOI; #, significantly decreased level of corresponding cytokine compared to *P. gingivalis* infection at a similar MOI. Exact *p*-values for all comparisons are provided in Supplementary File S1.

The production of pro-inflammatory cytokines by macrophages infected during M1 polarization is shown in Figure 6. *Fusobacterium nucleatum* and *T. forsythia* increased the production of most characteristic M1 cytokines, TNF-α, IL-1β, IL-6, IL-23, IL-12p40, and IL-12p70, whereas IFN-γ and IP-10 were unaffected. *Porphyromonas gingivalis* significantly increased IL-1β, IFN-γ, and IL-23; significantly decreased IP-10 and left the remaining cytokines unchanged. Notably, IFN-γ reached its highest level at the lowest *P. gingivalis* MOI of 25 and declined at higher MOIs. With the exception of IFN-γ, all cytokine levels were significantly lower after infection with *P. gingivalis* than with the other two bacteria at comparable MOIs, whereas IFN-γ levels were significantly higher.

**Figure 7:**
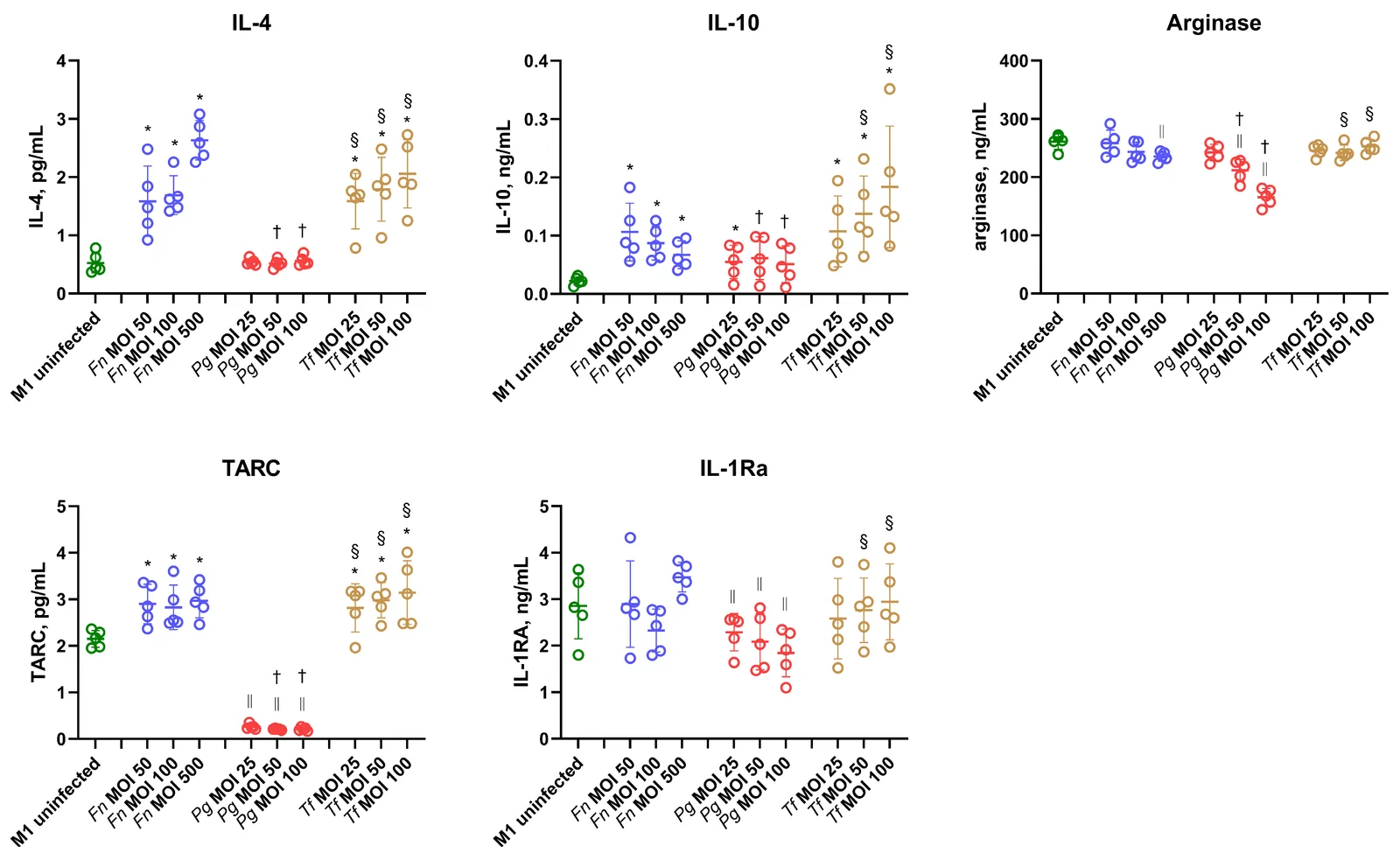
Anti-inflammatory cytokine production by M1 macrophages upon bacterial infection. THP-1 monocytes were differentiated into M0 macrophages by PMA treatment, then polarized toward the M1 phenotype by treatment with IFN-γ and *E. coli* LPS while being simultaneously infected with *F. nucleatum* (*Fn*), *P. gingivalis* (*Pg*), or *T. forsythia* (*Tf*) at different MOIs for 48 h. Levels of cytokines characteristic of M2 macrophages—IL-4, IL-10, and IL-1Ra—as well as arginase and TARC, in conditioned media were determined by multiplex assay. Y-axes show the corresponding mediator concentration in the conditioned media. Each data point represents an individual experiment; lines and error bars indicate the mean and SD, respectively. Symbols indicate statistically significant differences (*p* < 0.05). *, significantly increased level of corresponding cytokine compared with the uninfected control; ‖, significantly decreased level of corresponding cytokine compared to the uninfected control; †, significantly decreased level of corresponding cytokine compared with *F. nucleatum* infection at a similar MOI; §, significantly increased level of corresponding cytokine compared with *P. gingivalis* infection at a similar MOI. Exact *p*-values for all comparisons are provided in Supplementary File S1.

The production of anti-inflammatory cytokines by macrophages infected during M1 polarization is shown in Figure 7. *Fusobacterium nucleatum* and *T. forsythia* increased the levels of IL-4, IL-10, and TARC, but not of arginase or IL-1Ra. *Porphyromonas gingivalis* significantly decreased arginase, TARC, and IL-1Ra at most MOIs and slightly increased IL-10 at an MOI of 25. Levels of IL-4, arginase, and TARC were significantly higher after infection with *F. nucleatum* and *T. forsythia* than with *P. gingivalis* at comparable MOIs, and *T. forsythia* induced higher IL-1Ra levels than *P. gingivalis*.

### 3.4. Effect of Bacterial Infection on the Characteristic Surface Marker Expression and Cytokine Production in M2 Macrophages

**Figure 8:**
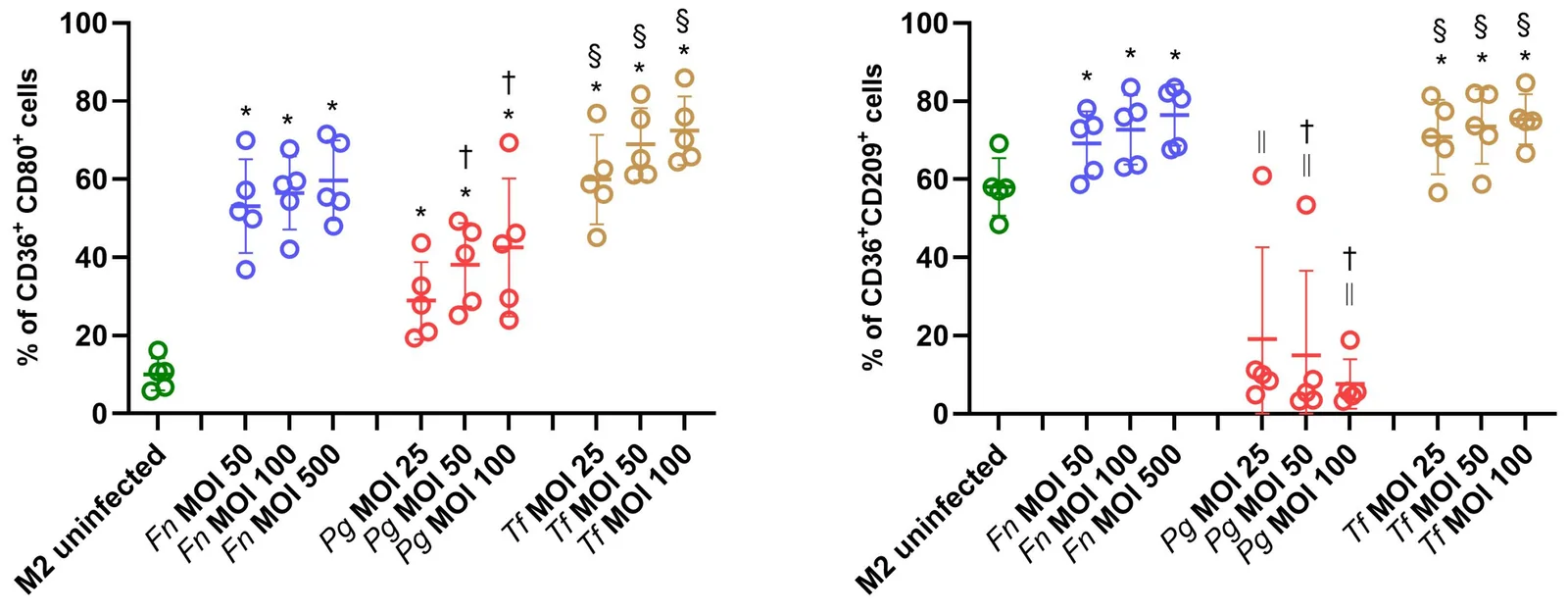
Surface marker expression of M2 macrophages upon bacterial infection. THP-1 monocytes were differentiated into macrophages by PMA treatment and polarized toward the M2 phenotype by treatment with IL-4 and IL-13, in the presence or absence of *F. nucleatum* (*Fn*), *P. gingivalis* (*Pg*), or *T. forsythia* (*Tf*) at different MOIs. Expression of the characteristic M1 and M2 surface markers CD80 and CD209, respectively, was analyzed by flow cytometry; staining for CD36 confirmed macrophage differentiation. Y-axes show the percentage of CD36^+^CD80^+^ cells and CD36^+^CD209^+^ cells, corresponding to M1 and M2 macrophages, respectively. Each data point represents an individual experiment; lines and error bars indicate the mean and SD, respectively. Symbols indicate statistically significant differences (*p* < 0.05). *, significantly increased % of CD36^+^CD80^+^ or CD36^+^CD209^+^ cells compared to the uninfected control; ‖, significantly decreased % of CD36^+^CD80^+^ or CD36^+^CD209^+^ cells compared to the uninfected control; †, significantly decreased % of CD36^+^CD80^+^ or CD36^+^CD209^+^ cells compared with *F. nucleatum* infection at a similar MOI; §, significantly increased % of CD36^+^CD80^+^ or CD36^+^CD209^+^ cells compared with *P. gingivalis* infection at a similar MOI. Exact *p*-values for all comparisons are provided in Supplementary File S1.

The surface expression of CD80 and CD209 in THP-1 macrophages infected during M2 polarization is shown in Figure 8. The proportion of CD80^+^ macrophages increased with all three bacteria at all tested MOIs. However, at comparable MOIs, the increase induced by *P. gingivalis* was significantly smaller than that induced by *F. nucleatum* and *T. forsythia*. The proportion of CD209^+^ macrophages was significantly increased by *F. nucleatum* and *T. forsythia* and significantly decreased by *P. gingivalis*.

**Figure 9:**
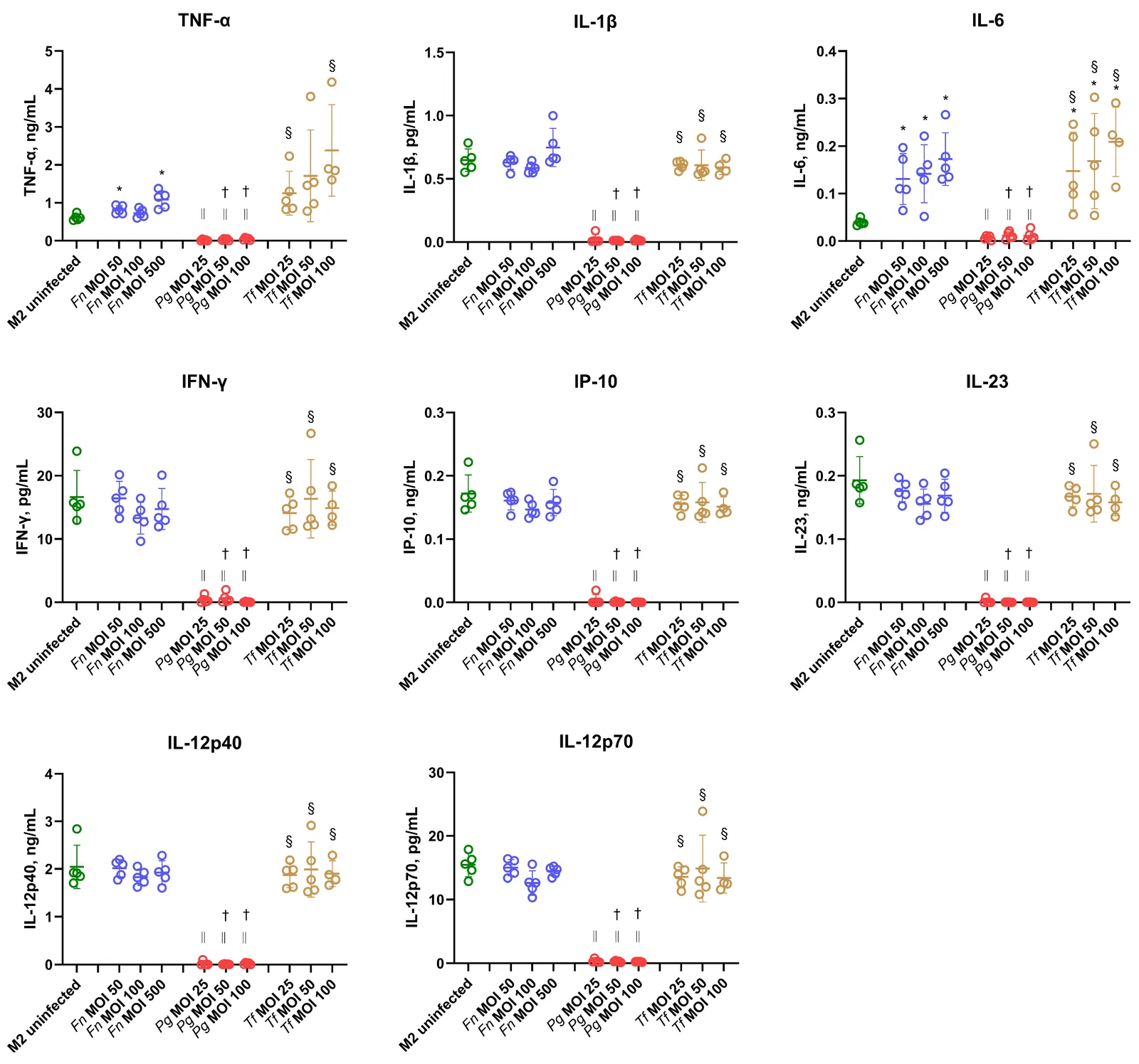
Pro-inflammatory cytokine production by M2 macrophages upon bacterial infection. THP-1 monocytes were differentiated into macrophages by PMA treatment and polarized toward the M2 phenotype by treatment with IL-4 and IL-13. Polarization was performed in the presence or in the absence of *F. nucleatum* (*Fn*), *P. gingivalis* (*Pg*), or *T. forsythia* (*Tf*) at different MOIs. Levels of cytokines characteristic of M1 macrophages—IL-1β, TNF-α, IL-6, IFN-γ, IP-10, IL-23, IL-12p40, and IL-12p70—in conditioned media were determined by multiplex assay. Y-axes show the corresponding cytokine concentration in the conditioned media. Each data point represents an individual experiment; lines and error bars indicate the mean and SD, respectively. Symbols indicate statistically significant differences (*p* < 0.05). *, significantly increased level of corresponding cytokine compared to the uninfected control; ‖, significantly decreased level of corresponding cytokine compared with the uninfected control; †, significantly decreased level of corresponding cytokine compared with *F. nucleatum* infection at a similar MOI; §, significantly increased level of corresponding cytokine compared with *P. gingivalis* infection at a similar MOI. Exact *p*-values for all comparisons are provided in Supplementary File S1.

**Figure 10:**
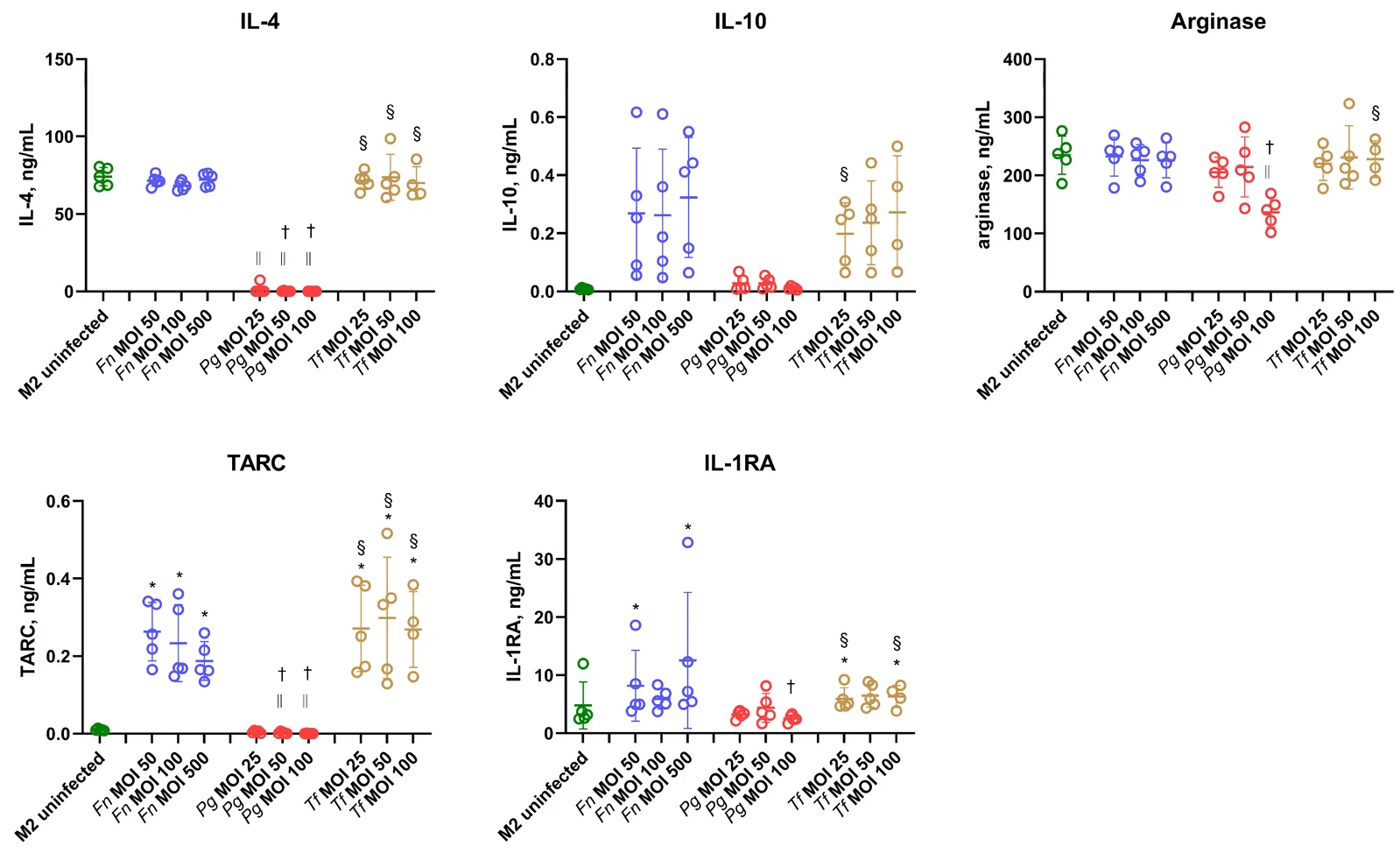
Anti-inflammatory cytokine production by M2 macrophages upon bacterial infection. THP-1 monocytes were differentiated into M0 macrophages by PMA treatment and polarized toward the M2 phenotype by treatment with IL-4 and IL-13. Polarization was performed in the presence or in the absence of *F. nucleatum* (*Fn*), *P. gingivalis* (*Pg*), or *T. forsythia* (*Tf*) at different MOIs. Levels of cytokines characteristic of M2 macrophages—IL-4, IL-10, and IL-1Ra—as well as arginase, and TARC in conditioned media were determined by multiplex assay. Y-axes show the corresponding cytokine concentration in the conditioned media. Each data point represents an individual experiment; lines and error bars indicate the mean and SD, respectively. Symbols indicate statistically significant differences (*p* < 0.05). *, significantly increased level of corresponding cytokine compared with the uninfected control; ‖, significantly decreased level of corresponding cytokine compared with the uninfected control; †, significantly decreased level of corresponding cytokine compared with *F. nucleatum* infection at a similar MOI. §, significantly increased level of corresponding cytokine compared with *P. gingivalis* infection at a similar MOI. Exact *p*-values for all comparisons are provided in Supplementary File S1.

The production of pro-inflammatory cytokines by macrophages infected during M2 polarization is shown in Figure 9. *Fusobacterium nucleatum* significantly increased TNF-α and IL-6, and *T. forsythia* significantly increased IL-6, whereas the remaining detectable cytokines, IL-1β, IFN-γ, IP-10, IL-23, IL-12p40, and IL-12p70, were unaffected by either species. In contrast, *P. gingivalis* decreased the levels of all characteristic M1 cytokines, which were also significantly lower than those observed with the other two bacteria at comparable MOIs. The production of anti-inflammatory cytokines by macrophages infected during M2 polarization is shown in Figure 10. *Fusobacterium nucleatum* and *T. forsythia* increased the levels of IL-4, IL-10, and TARC, but not of arginase or IL-1Ra. *Porphyromonas gingivalis* significantly decreased arginase, TARC, and IL-1Ra at most tested MOIs and slightly increased IL-10 at an MOI of 25. Levels of IL-4, arginase, and TARC were significantly higher after infection with *F. nucleatum* and *T. forsythia* than with *P. gingivalis* at comparable MOIs, and *T. forsythia* induced higher IL-1Ra levels than *P. gingivalis*.

## 4. Discussion

In this study, we examined how infection with periodontitis-associated bacteria influences human macrophage polarization and cytokine production. Using THP-1-derived macrophages as a host cell model, we assessed the in vitro response to bacterial infection under several conditions: infection of unpolarized M0 macrophages, and infection during polarization toward the M1 or M2 phenotype induced by IFN-γ plus LPS or IL-4 plus IL-13, respectively. As readouts, we measured macrophage viability, the proportions of cells expressing the M1-associated marker CD80 or the M2-associated marker CD209, and extracellular concentrations of a panel of pro- and anti-inflammatory cytokines by multiplex assay [25].

Bacterial infection generally did not compromise macrophage viability under any condition, except for *F. nucleatum* at an MOI of 500 during M1 polarization, where more than 80% of macrophages remained viable. This modest reduction may reflect the infection itself together with the high levels of pro-inflammatory cytokines produced under these conditions, particularly IL-1β, which can trigger apoptosis in various cell types [48]. Conversely, the elevated IL-1β production may partly be a consequence of increased cell death, since macrophage death is known to be accompanied by IL-1β release [49,50].

We observed no substantial effect of *P. gingivalis* or *T. forsythia* on THP-1-derived macrophage viability under any condition, which contrasts with previous reports [34,51]. One study found that infecting THP-1 macrophages with *P. gingivalis* or *T. forsythia* at MOIs of 10–500 induced pyroptotic cell death [51], whereas another showed that *P. gingivalis* at MOIs 50–100 triggered PANoptosis in murine bone marrow-derived macrophages [34]. These discrepancies may reflect differences in macrophage origin and experimental protocols. Notably, *P. gingivalis* has been reported to induce apoptosis in gingival fibroblasts [52,53] but to inhibit apoptosis in oral epithelial cells [54,55], suggesting that its pro-apoptotic activity is cell-type specific.

In M0 macrophages, infection with *F. nucleatum* and *T. forsythia* enhanced M1-associated responses, as indicated by an increased proportion of CD80^+^ cells and enhanced pro-inflammatory cytokine production, with no qualitative differences between the two species. *Porphyromonas gingivalis* elicited a different response: the increase in CD80^+^ cells was modest and not statistically significant, pro-inflammatory mediators were not elevated relative to uninfected controls, and IP-10 was reduced. Anti-inflammatory mediator levels in M0 macrophages were low and largely unaffected by infection, although *F. nucleatum* and *T. forsythia* increased IL-10 and *P. gingivalis* reduced arginase. Thus, in M0 macrophages, *F. nucleatum* and *T. forsythia* produced similar responses and enhanced M1-associated features, whereas *P. gingivalis* caused only weak activation.

Qualitative and quantitative differences between the bacteria were also evident during M1-associated polarization. Treatment with IFN-γ and LPS increased the proportion of CD80^+^ macrophages to about 80% (Figure 2 and Figure 5), and *F. nucleatum* and *T. forsythia* raised this proportion further. Both species also enhanced the production of nearly all measured pro-inflammatory cytokines except IFN-γ; the only difference between them was higher IL-6 and IL-23 induction by *T. forsythia* than by *F. nucleatum* at equal MOIs. In addition, both bacteria increased certain anti-inflammatory cytokines, notably IL-4, IL-10, and TARC. *P. gingivalis* again behaved differently: it reduced the proportion of CD80^+^ macrophages, only weakly induced pro-inflammatory cytokines—whose levels, except IFN-γ, were generally lower than with the other bacteria—and either suppressed or did not affect anti-inflammatory cytokine production. This weakened M1-associated response to *P. gingivalis* agrees with a recent study reporting that *P. gingivalis* suppresses the IFN-γ-induced response of THP-1 macrophages, as shown by RNA-seq and qPCR, through the action of proteases and extracellular vesicles [30].

Among the pro-inflammatory cytokines secreted during M1-associated polarization, IFN-γ responded to infection in a distinctive manner. Because IFN-γ was added to induce M1 polarization, its levels in conditioned media must be interpreted with caution. Nevertheless, *P. gingivalis* at the lowest tested MOI of 25 further increased IFN-γ, *F. nucleatum* increased it only at the very high MOI of 500, and *T. forsythia* had no effect. Intriguingly, higher *P. gingivalis* MOIs led to progressively lower IFN-γ levels. At the same time, IP-10, whose expression is driven by IFN-γ [54], was markedly reduced by *P. gingivalis* at all MOIs. This observation is consistent with a recent report showing that *P. gingivalis* attenuates IFN-γ-induced gene expression in THP-1 macrophages [30]. Together, these results suggest that *P. gingivalis* increases IFN-γ production even at low MOI while suppressing downstream IFN-γ signaling. The significance of this dual effect remains unclear. IFN-γ is a pro-inflammatory cytokine with potent antiviral activity [56] that also enhances the antimicrobial function of macrophages [57,58]; its inactivation by *P. gingivalis* could further contribute to immune subversion and the promotion of dysbiosis.

Infection during M2-associated polarization produced bacterium-dependent outcomes. As in M0 and M1-associated macrophages, *P. gingivalis* differed qualitatively from *F. nucleatum* and *T. forsythia*. All three species increased the proportion of CD80^+^ macrophages, but the effect of *P. gingivalis* was weakest, and only *P. gingivalis* reduced the proportion of CD209^+^ macrophages. *Porphyromonas gingivalis* decreased all pro-inflammatory cytokines, whereas the other two species left them unchanged or increased them. Likewise, *F. nucleatum* and *T. forsythia* maintained or increased anti-inflammatory cytokine concentrations, whereas *P. gingivalis* reduced three of the five measured mediators—IL-4, arginase, and TARC. As IL-4 was added at 20 ng/mL to induce polarization, its reduction to a few pg/mL in the presence of *P. gingivalis* may reflect proteolytic degradation [59]. The concurrent increase in both pro- and anti-inflammatory cytokines, together with increased CD80^+^ and CD209^+^ proportions following infection with *F. nucleatum* and *T. forsythia*, highlights the limitations of a strict M1/M2 classification. Such responses may reflect inflammatory activation accompanied by regulatory feedback mechanisms; for instance, increased IL-10 may act to limit inflammation rather than indicate a separate M2-polarization program [60]. The combined cytokine profiles may also reflect heterogeneity within the macrophage population [61]. These findings therefore support the view of macrophage activation as a spectrum of context-dependent, overlapping responses rather than discrete M1 and M2 polarization states [62]. Overall, our data indicates that *P. gingivalis* suppresses M2-associated polarization of THP-1 macrophages, whereas *F. nucleatum* and *T. forsythia* slightly enhance it while concurrently shifting the cells toward an M1-associated phenotype. This finding regarding *P. gingivalis* is consistent with an earlier report in mouse macrophages [31].

Taken together, our findings lead to the clear conclusion that, under all tested conditions, macrophages respond to *P. gingivalis* differently from *F. nucleatum* and *T. forsythia*. *Porphyromonas gingivalis* inhibited both M1- and M2-associated polarization and reduced cytokine production by the macrophages. This distinct behavior may result from multiple mechanisms by which *P. gingivalis* subverts host responses [29,63], as well as from the proteolytic degradation of host cytokines by gingipains [59,64,65]. Inhibition of M1 polarization could blunt the pro-inflammatory response required to eliminate invading pathogens and control the infection, while inhibition of M2 polarization may impair inflammation resolution and delay tissue regeneration. These effects are fully consistent with the role of *P. gingivalis* as a keystone pathogen within the subgingival microbial community [14]. By contrast, *F. nucleatum* and *T. forsythia* elicited qualitatively similar responses: both strongly promoted pro-inflammatory mediator production during M1-associated polarization and exerted a moderate pro-inflammatory effect in M0 macrophages and during M2-associated polarization. This pro-inflammatory stimulation aligns with previous reports [36,66] and with the proposed role of *F. nucleatum* and *T. forsythia* as pathobionts that amplify inflammation and promote dysbiosis [12,16,19].

Macrophages are the resident cells of the gingiva and are present even in health [67]. The relationship between macrophage phenotype and the transition from health to gingivitis and then to periodontitis is complex. Although shifts in macrophage polarization have been detected during disease, no clear pattern has emerged [67,68]. Even so, the properties of the different phenotypes can be tentatively translated into *in vivo* function. M0 macrophages are unpolarized cells in a basal state [69] that may act as sentinels guarding healthy gingiva against invading pathogens. M1 macrophages produce large amounts of pro-inflammatory cytokines [23,24] and may be pivotal during disease initiation and active phases, whereas M2 macrophages are likely involved in resolution and tissue repair. Integrating our data with this framework, we propose that *P. gingivalis* disrupts pathogen elimination, inflammation resolution, and tissue regeneration by macrophages, whereas *F. nucleatum* and *T. forsythia* intensify inflammation during active disease.

The principal limitation of this study is its in vitro model, in which a macrophage cell line was challenged with individual bacterial species. THP-1 cells, derived from a child with acute leukemia [70], do not fully recapitulate gingival tissue-resident macrophages, and IFN-γ/LPS- or IL-4/IL-13-induced polarization does not fully reproduce the spectrum of macrophage states in vivo. Phenotypic characterization relied on a single surface marker per polarization condition; although cytokine profiles provided additional information, these measurements do not allow a definite assignment of M1 or M2 states. Because bacteria were added during polarization, our findings cannot distinguish effects on the development of polarization-associated features from concurrent modulation of macrophage responses. Moreover, single-species infections with one strain per species do not capture subgingival multispecies biofilm–host interactions or strain-dependent variability—for example, certain lactobacilli have recently been shown to attenuate the pro-inflammatory and proteolytic activity of *P. gingivalis* [71]. As bacterial viability at 48 h was not assessed, differences in bacterial viability may have contributed to the observed macrophage responses. Finally, cytokine measurements were restricted to culture supernatants, without mRNA analysis or direct analysis of cytokine degradation, which prevented us from distinguishing changes in cellular production from potential proteolytic effects. Future studies using primary human macrophages with expanded phenotypic characterization, and multispecies biofilm models incorporating multiple strains per species are needed to confirm and extend these findings.

## 5. Conclusions

Our findings show that periodontitis-associated bacteria exert strikingly different effects on human macrophage polarization. *Porphyromonas gingivalis* suppressed both M1- and M2-associated polarization and dampened cytokine production, consistent with its role as a keystone pathogen that subverts the innate immune response and thereby impairs pathogen clearance, inflammation resolution, and tissue repair. In contrast, *F. nucleatum* and *T. forsythia* amplified the pro-inflammatory response and drove macrophages toward the M1-associated phenotype, in line with their roles as periodontal pathobionts that augment inflammation. Together, these species-specific responses provide mechanistic insight into how individual members of the subgingival community contribute to the host-microbiome imbalance underlying periodontitis, and may inform strategies aimed at restoring immune homeostasis.

## Data Availability

The original data presented in the study are openly available in Zenodo at https://doi.org/10.5281/zenodo.23060367.

## Supplementary Materials

The following supporting information can be downloaded at: https://www.mdpi.com/article/10.3390/cells15212016/s1, Supplementary Figure S1: Gating strategy for M1 and M2 macrophage identification upon bacterial infection. Flow cytometry was performed on an Attune NxT flow cytometer, and data were analyzed with FCS Express^TM^ 7. **A:** Cells were gated for singlets using Side Scatter Area (SSC-A) and Side Scatter Width (SSC-W). **B:** Cell debris was excluded by gating Forward Scatter Area (FSC-A) against SSC-A. **C:** FVD eFluor780 was used to discriminate between living and dead cells. **D:** Successfully matured macrophages were assessed by gating on CD36^+^ events. **E:** CD36^+^ events were gated for CD36^+^CD80^+^ events reflecting M1 polarization. **F:** CD36^+^ events were gated for CD36^+^CD209^+^ events reflecting M2 polarization. Supplementary File S1: Exact *p*-values for all comparisons.

## Abbreviations

The following abbreviations are used in this manuscript:

CBA	Columbia Blood Agar
CFU	colony-forming units
FA	Fastidious Anaerobe agar
FBS	fetal bovine serum
FSC	Forward Scatter
FVD	Fixable Viability Dye
*F*. *nucleatum*	*Fusobacterium nucleatum*
IFN	interferon
IL	interleukin
IL-1Ra	IL-1 receptor antagonist
IP	IFN-γ-induced protein
LPS	lipopolysaccharide
M0	unpolarized macrophages
MOIs	multiplicities of infection
NAMA	*N*-acetylmuramic acid
OD_600_	optical density at 600 nm
*P. gingivalis*	*Porphyromonas gingivalis*
PBS	phosphate-buffered saline
PMA	phorbol-12-myristate-13-acetate
SSC	Side Scatter
SSC-A	Side Scatter Area
SSC-W	Side Scatter Width
*T. denticola*	*Treponema denticola*
*T. forsythia*	*Tannerella forsythia*
TARC	thymus and activation-regulated cytokine
TNF	tumor necrosis factor
