# Supplementary material for: Distinct Polarization of Human Macrophages in Response to Infection with the Periodontitis-Associated Bacteria Fusobacterium nucleatum, Porphyromonas gingivalis, and Tannerella forsythia
Source: Cells. 2026 Nov 5;15(21):2016. doi: 10.3390/cells15212016 (PMC13762304; doi:10.3390/cells15212016)
Supplement: Supplementary file 1 [file cells-15-02016-s001.zip › cells-4631504-Supplementray Figure S1.pdf]

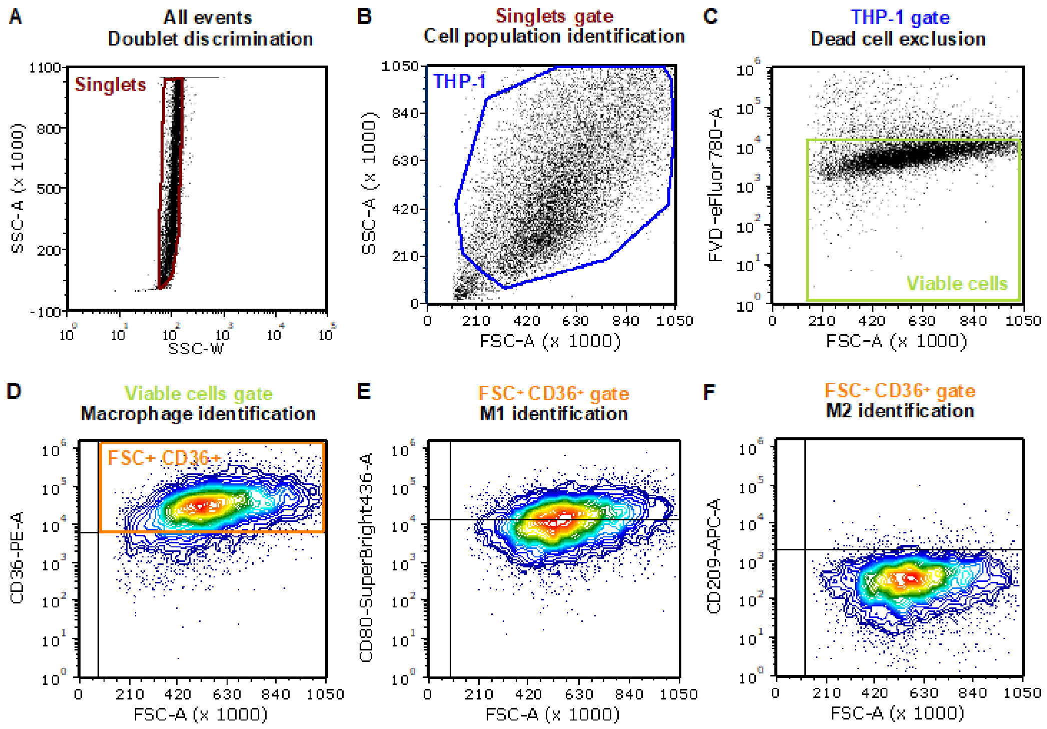

Supplementary Figure S1. Gating strategy for M1 and M2 macrophage identification upon bacterial infection. Flow cytometry was performed on an Attune NxT flow cytometer, and data were analyzed with FCS Express™ 7. **A:** Cells were gated for singlets using Side Scatter Area (SSC-A) and Side Scatter Width (SSC-W). **B:** Cell debris was excluded by gating Forward Scatter Area (FSC-A) against SSC-A. **C:** FVD eFluor780 was used to discriminate between living and dead cells. **D:** Successfully matured macrophages were assessed by gating on CD36^{+} events. **E:** CD36^{+} events were gated for CD36^{+}CD80^{+} events reflecting M1 polarization. **F:** CD36^{+} events were gated for CD36^{+}CD209^{+} events reflecting M2 polarization.
